# Effects of salient factors on the pursuit of higher education among multicultural youth in Hong Kong

**DOI:** 10.3389/fpsyg.2023.1253842

**Published:** 2023-12-07

**Authors:** Celeste Y. M. Yuen, Alan C. K. Cheung, K. H. Leung

**Affiliations:** ^1^Department of Educational Administration and Policy, Faculty of Education, The Chinese University of Hong Kong, Shatin, Hong Kong SAR, China; ^2^The Hong Kong Centre for the Development of Educational Leadership, The Chinese University of Hong Kong, Shatin, Hong Kong SAR, China

**Keywords:** educational functionalism beliefs, higher education, underprivileged mainstream students, Chinese immigrant students, South Asian students, Hong Kong

## Abstract

This study presents novel and compelling evidence of the disparities in factors influencing the beliefs and aspirations for higher education among mainstream and immigrant youth in Hong Kong, particularly those who are underprivileged. We developed and validated a psychometric questionnaire, known as the Post-Secondary Education Pursuit Instrument (PSEPi), which was administered to 4,850 students aged between 15 and 18 years old from 23 secondary schools. The objective of this study was to explore the factors that impact students’ choices and plans for higher education. The results of the one-way Multivariate Analysis of Variance (MANOVA) analysis deepen our understanding of the differential effects of success and obstacle factors on students’ higher education pursuits across various cultural groups. The underprivileged mainstream, Chinese immigrant, and ethnic minority South Asian youth reported a perceived glass ceiling effect associated with their ethnic backgrounds, as well as financial aid barriers, while pursuing higher education, in contrast to their average Hong Kong mainstream counterparts (mean Cohen’s *d* = 0.40). The direct effects and multiple mediation analyses demonstrated that significant others’ influence, particularly the influence of parents, and locus of control are prime determinants of the perceived usefulness of higher education for all student groups. The implications are that educational policies should be implemented to level the playing field in higher education admissions for both privileged and underprivileged youth in Hong Kong and other international countries. Overall, this study provides robust empirical evidence that can be utilized to enhance educational policies and practices to bridge the gap between mainstream and underprivileged immigrant youth in their pursuit of higher education.

## 1 Introduction

Access to higher education is a crucial aspect of upward social mobility and personal growth (Sandmann et al., 2019). The journey toward higher education starts in secondary school and intensifies in senior secondary education. However, researchers (Kim, 2014; Sandmann et al., 2019) note that the institutional hierarchies within higher education often perpetuate social inequalities. The expansion of higher education has only widened the gap between privileged and underprivileged students, with the latter still struggling to access these institutions. In England, despite recent efforts to increase access to higher education, disadvantaged students from low socio-economic backgrounds continue to face significant barriers (Chowdry et al., 2011).

Higher education has expanded significantly in Asia and brought a more diverse range of programs and students. However, taking China as an example, higher education choices and fee structures still reflect social stratification, making it difficult to achieve social inclusion and educational equality (Ding et al., 2021). This is a global issue that requires urgent attention to remove the socio-economic and financial barriers that prevent underprivileged youth from accessing, proceeding, and completing their studies successfully (Nutt and Hardman, 2019). Previous research (Dave et al., 2019; Kalu, 2022; Refaeli et al., 2023) has shown that students’ higher education choice hinges on multiple factors, including personal factors (academic capability and self-esteem) and social factors (family socio-economic status, parental expectation, school quality, and resources). The transition from senior secondary school to higher education is complex and affected by individual and social factors, some of which are within and outside of an individual’s control (O’Donnell et al., 2016), calling for creating favorable conditions for different minority groups. The Combined model for higher education choice highlights the importance of personal and contextual factors in determining higher education aspirations (Hossler et al., 1999). Essentially, the combined model underscores the prime roles of the individual (e.g., academic ability) and social (e.g., social capital, parental support) factors in determining higher education aspiration. Several antecedents of higher education pursuits, such as the perceived usefulness of education and family influence, have been well-documented in Western and Asian literature. Therefore, it is essential to consider these factors when developing policies and programs that aim to increase access to higher education for all students (Turner, 2018; Guan and Ploner, 2020; Sier, 2021; Abdalla et al., 2022).

### 1.1 Chinese immigrant and ethnic minority students in Hong Kong

Hong Kong was once regarded as a haven by Chinese immigrants (Chan and Chou, 2016) with around 90% being Han Chinese. The Chinese immigrants are heterogenous groups, with a wide range of backgrounds, dialects, customs, beliefs, and socio-economic status, and different from local Hong Kong. The significance of these factors cannot be underestimated when considering the experiences and challenges their children face in Hong Kong schooling. Research (Yuen, 2010; Yang et al., 2020) has shed light on the complex interplay between cultural identity, social status, and educational opportunities for Chinese immigrant students (CIS). CIS admitted to secondary schools grew steadily from 2644 in 2014/15 to 3804 but dropped sharply to 2340 in 2019/20 due to intercultural conflicts associated with social unrest. The pedagogical approach adopted by CIS has been known to elicit frustration among students (Yuen, 2022). The requirement to acquire fluency in the mainstream dialect of Cantonese, coupled with the need to transition from simplified to complex Chinese characters, are significant obstacles to learning. Additionally, the placement arrangements for overage students are one major impediment, as it often necessitates repeating 1–2 years of academic coursework to improve one’s English proficiency. Such policies can have the detrimental effect of eroding their academic self-esteem as teachers are significant figures, and their perceptions of model students shape the expectations and behaviors of students (Chee, 2012). Moreover, leaving their mainland peers leads to a sense of disconnectedness. Disjointed social networks and limited homework support from family members, both lead to underachievement (Pong, 2009; Yuen, 2022).

Ethnic minorities with South/Southeast origins in Hong Kong recorded a 120% increase between 2006 and 2016 (Census and Statistics Department, 2016). The South/Southeast Asian students (SAS) whose parents are from the Philippines, India, Nepal, and Pakistan are gaining attention regarding their access to higher education (Bhowmik, 2019; Keung and Ho, 2020; Te, 2020; Yuen, 2022). Sharing the frustrations of the assimilationist approach, SAS students face intersectionality challenges (Cho et al., 2013), in their pursuit of higher education. Race, religion, identity, and language are known factors that cause them to lead an isolated lifestyle and hold a stereotyped perception of society (Crabtree and Wong, 2013; Gao et al., 2019; Loh and Hung, 2020). Language proficiency defines failure and success. Many Hong Kong-born Pakistanis, Indians and Filipinos are native Cantonese speakers but have limited proficiency in the Chinese Language, impeding their academic performance (Loh and Hung, 2020; Yuen, 2022). A deeper understanding of the unique circumstances of these individuals is essential for developing policies and interventions that can effectively support their integration and well-being. While some studies have addressed systemic inequalities and structural barriers in the education system (Chee, 2012; Bhowmik, 2019; Gao et al., 2019; Te, 2020), only a few have examined the differential impacts of factors affecting higher education pursuits on different ethnic groups.

Since 2006, there has been a significant upsurge in post-secondary education (PSE) in Hong Kong. Notably, the offering of self-financed 2-year sub-degree programs, such as higher diplomas and sub-degrees, has experienced substantial growth, providing access to over 60% of the HKDSE graduates (Kember, 2010). Between 2000 and 2010, student enrollments in sub-degree programs increased from 9,549 to 34,949, surpassing the increase in degree program enrollments, which only rose from 14,209 to 18,766. Currently, 22 higher education institutions confer degrees, as well as 22 post-secondary institutions that offer sub-degree programs. These institutions afford a diverse range of study options, featuring multiple entry and exit points. Each institution is characterized by its own unique mission and vision, striving for geopolitical significance (Sandmann et al., 2019). However, Lo (2017) criticized the education hub strategy, contending that it dissuades underprivileged students from pursuing higher education due to concerns about student debt, intense competition from non-local students, and dim employment prospects following graduation. Meanwhile, Chiu and Siu (2022) claimed that the current neoliberalism of higher education in Hong Kong exacerbates the marginalization of ethnic minority students. Institutions are drivers for the marketization of education, which treats the purpose of learning as a commodity and exacerbates the disparities between the privileged and underprivileged in terms of education outcomes.

To address the prevalent social and educational inequalities within Hong Kong’s higher education system (Manning and Yuen, 2023), it is imperative to engage in collaborative institutional research endeavors that incorporate perspectives from diverse stakeholders representing various disciplines. This approach will foster synergistic efforts toward developing a comprehensive understanding of the systemic barriers confronted by underprivileged students. By generating a big dataset, valuable insights can be gleaned into the issues faced by these students, thereby facilitating the development of effective interventions aimed at enhancing their educational outcomes.

Existing research in this field has primarily relied on case studies and qualitative investigations (Bhowmik, 2019; Gao et al., 2019; Te, 2020). Hence, there is a pressing need for more comprehensive empirical evidence from quantitative studies that can provide a deeper understanding of the complex interplay of personal and social factors that shape the experiences of the entire student population (Queirós et al., 2017). This study is, hence, highly trendy and significant to fill these research gaps by employing a validated survey questionnaire to identify the prime success and obstacle factors influencing the three ethnic groups: mainstream students, Chinese immigrant students, and ethnic minority students to pursue higher education within Hong Kong. Furthermore, this study is novel to examine the mechanisms underlying the formation of educational functionalism beliefs that are associated with success and obstacle factors. Given its significant and timely nature, the findings of this research endeavor will shed new light for policy makers and educators, as to how to eliminate educational inequalities and promote inclusivity in higher education.

### 1.2 The conceptual models

The primary objective of this study is to scrutinize the crucial variables that influence students’ inclination and endeavor toward attaining higher education, particularly across diverse student groups. In order to accomplish these research aims, we have identified the factors espoused by the Combined model (Hossler et al., 1999) and Harris and Halpin’s (2002) model as instrumental in shaping our study. Both models share overlapping and complementary factors. For instance, the economic perspective of Hossler et al.’s (1999) model concentrates on the cost-benefit aspect of the financial burden and contributions of higher education to high-paying jobs and social status. This perspective overlaps with the relative functionalism factor advocated by Harris and Halpin (2002), or the instrumental values of education, compared to other non-educational choices. Scholarships and financial aid are crucial in enabling low-income students to attend college and cultivate their talent. The sociological model analyses the interaction of sociological and psychological factors in terms of financial feasibility and individuals’ academic ability, when determining whether to pursue higher education or not (Hossler et al., 1999). Students weigh economic and sociological factors when continuing education beyond high school. They research different institutions, gather information, and enroll in the ones that best meet their needs. Self-determination and perceived ability have a direct impact on academic aspiration. Family influences mediate their attitudes toward the value of higher education. Hence, providing sufficient financial aid and scholarships can increase the chance of lower-income students to pursue higher education.

Harris and Halpin’s (2002) model offers a comprehensive approach that complements Hossler et al.’s (1999) work by examining economic, social, cultural, psychological factors, and educational processes in pursuit of a holistic understanding of the factors that motivate individuals to pursue higher education. The two models highlight the importance of job opportunities, social status, income, and prestige as key motivators for higher education enrollment. This study, guided by Harris and Halpin’s (2002) model, focuses on seven primary factors, including significant others’ influence, glass ceiling, relative functionalism, locus of control, self-efficacy, preparation for college, and financial aid among different student groups. An in-depth analysis of each of these factors will be presented below.

#### 1.2.1 Significant others’ influence

The influence exerted by parental expectations, warmth, and financial support is one critical determinant of students’ educational aspirations and motivation. Family matters. Bourdieu et al.’s (1990) capital theory underscores that family is a valuable social capital and a habitus for the privileged to situate their children in a conducive learning environment to form their aspirations for future success. Compared to working-class parents, middle-class parents subscribe to school expectations to arm their children with the essential skills and knowledge for higher education and vertical social mobility (Lareau, 2011). The capital theory offers a vital lens to uncover the thought process behind Chinese youth’s aspirations for higher education. Influenced by the Confucian heritage culture in Hong Kong (Katyal and King, 2011), the strong family-oriented culture and moral values of filial piety, deference, kinship ties, self-efficacy, and academic success to honor parents are systematically instilled in their children through family socialization. In contemporary Chinese society, good parenting is primarily about assisting children in academic success, which often translates into pursuing higher education and obtaining high-income jobs (Xiang and Chiu, 2022). The concerted cultivation approach to maximizing the opportunity for children’s academic success is phenomenal (Ho et al., 2018). Singaporean students’ educational aspirations are closely related to their parents’ socio-economic status, which is a typical example (Ng and Choo, 2021).

#### 1.2.2 Glass ceiling

The glass ceiling effect is a term used to describe the societal obstacles that impede the career advancement of women and people of color based on their gender and race (Jackson et al., 2014). In the context of education, students from marginalized backgrounds, such as those who are deprived, immigrants, or belong to ethnic minority groups, face various barriers that hinder their academic progress (Chowdry et al., 2013). Research has indicated that a significant number of students encounter the glass ceiling effect, which is a phenomenon that arises when they perceive a lack of adequate preparation, knowledge, resources, and confidence to pursue their desired paths of higher education (Harris, 2015; Yuen et al., 2021). A critical factor leading to lower participation rates in higher education is the lower attainment rate of secondary education. The relationship between academic performance and educational aspirations is complex. Chowdry et al. (2011) argue that increasing the aspirations of students from lower socio-economic backgrounds alone is unlikely to eliminate the socio-economic differences in higher education participation. In Hong Kong, the glass ceiling effect is prevalent among low-income mainstream Hong Kong Chinese, Chinese immigrants, and ethnic minority Hong Kong youth (Yuen et al., 2021). These groups face the challenge of low levels of self-efficacy belief, limited support from parents and teachers, and inadequate financial assistance to realize their higher education aspirations. Consequently, they remain an underrepresented group in higher education institutions.

#### 1.2.3 Relative functionalism

This term refers to students’ belief in and subjective assessment of the functions and usefulness of education in generating cultural and socio-economic capital for individuals and families compared to alternative pathways (Sue and Okazaki, 1990; Harris and Halpin, 2002; Coy-Ogan, 2009). Research has demonstrated that the significance of a particular life choice holds a pivotal role in shaping students’ aspirations for higher education (Harris and Halpin, 2002; Coy-Ogan, 2009), which subsequently motivates them to pursue it further (Sier, 2021; Bataeineh, 2022; Vietze et al., 2022). The usefulness of higher education includes the acquisition of career prospects, vertical mobility, improved life chances, and social status, which are among the benefits of pursuing higher education, as posited by Bourdieu et al. (1990). Furthermore, higher education has been noted by Sue and Okazaki (1990) to promote greater self-efficacy due to its relative functionalism. However, it is important to recognize that students from diverse ethnic backgrounds may ascribe varying degrees of value to higher education. Turcios-Cotto and Milan’s (2013) study, for instance, revealed that Latino students tend to view higher education as a means of achieving individuality and material gain more so than their White counterparts.

#### 1.2.4 Self-efficacy and locus of control

This term refers to students’ perception of self-efficacy and perceived control over their educational goals and motivation to pursue higher education (Lawrence and Nkoane, 2020). The issue of negative racial stereotypes faced by ethnic minority students from dominant groups has been extensively researched in academic literature (Ku, 2006; Crabtree and Wong, 2013; Brown et al., 2017; Sier, 2021; Abdalla et al., 2022). These stereotypes can have a detrimental effect on the self-perception and academic success of minority students, leading to a reduced sense of control over their educational outcomes and a lack of motivation to pursue higher education (Brown et al., 2017). Conversely, when disadvantaged students feel empowered and in control of their academic success, they are more likely to value education and be incentivized to pursue further studies (Nordstrom and Segrist, 2009). Recent studies conducted in Hong Kong have shown that low-income students with positive academic self-efficacy beliefs are better equipped to build resilience and inner strength, which in turn helps them in their pursuit of higher education (Yuen, 2022). These findings highlight the importance of addressing negative stereotypes and empowering students to take control of their academic success (Lawrence and Nkoane, 2020). Conversely, disadvantaged students may perceive limited opportunities for accessing quality education compared to their mainstream counterparts, as evidenced by international studies (Reay et al., 2001). Negative beliefs can discourage working-class students from aspiring to attend university (Hutchings and Archer, 2001). Research suggests negative beliefs can deter working-class students from pursuing higher education (Hutchings and Archer, 2001). Additionally, ethnic minority students may face detrimental racial stereotypes that diminish their sense of control over academic achievement (Brown et al., 2017). However, fostering internal control over academic success can inspire disadvantaged students to value education and strive for higher learning opportunities (Nordstrom and Segrist, 2009). Moreover, positive academic self-efficacy beliefs can empower low-income students to develop resilience and inner strength to pursue higher education (Yuen, 2022).

#### 1.2.5 Preparation for college

Access to program information is a critical factor for students when determining which college to attend. Nowadays, college preparation has emerged as a key policy priority, and many parents have turned to expert consultants to help guide their children through the complex pathways of higher education. Schools offer academic mentoring services, organize campus tours to post-secondary institutions, and host seminars and talks on university program applications. These programs have become increasingly essential in preparing high school students for the rigors of college enrollment. For example, according to Gale and Parker (2014), students who are the first in their families to attend college experience a significant cultural shift between secondary school and college regarding learning and pedagogy. Consequently, college induction programs have become a pivotal component in assisting these students in navigating the complex pathways of higher education. Past research (Yuen, 2022) has demonstrated that low-income mainstream students in Hong Kong, Chinese immigrant students, and South Asian students face more significant obstacles than their mainstream counterparts in pursuing higher education due to limited access to college program information, language barriers, and inadequate financial support.

#### 1.2.6 Financial aid

The question of college costs has regained attention in academic discourse. The provision of formal financial assistance, such as bursaries, studentships, or scholarships, can assist underprivileged students in covering their tuition fees and is a crucial factor in promoting their enrollment in college. However, in the current era of marketization and neo-liberalism, higher education is increasingly viewed as a commodity, and the availability of financial aid can significantly impact the access of socially disadvantaged students to higher education (Woodall et al., 2014). As a result, the expansion of higher education has exacerbated class-related educational and employment disparities.

#### 1.2.7 Interrelationship among success and obstacle factors affecting higher education pursuit

Beliefs in the function of higher education and the broader societal values of education are shaped by personal, educational experiences and culture (Gorard et al., 1998). Addi-Raccah and Israelashvili (2014) investigated the effect of a university outreach program on the enrollment of low-income students in higher education. They concluded that increasing their personal capability would motivate them to pursue higher education. There seems to be a compensatory effect of personal capability on poverty. Literature has reiterated the role of significant others’ influence on students’ higher education aspirations. Parental care and teacher support are enablers to boost students’ academic self-efficacy and educational choices and planning (Wang and Neihart, 2015). Woelfel and Haller’s (1971) classic work on the impact of significant others and attitude formation among 100 high school seniors in the United States, theorized that interpersonal influence (significant others), self-reflexive activity (the categorization of the information), and related attitudes (valuable or not) toward objects have a strong influence on the attitude formation toward educational and occupational aspirations. Such influences are not linear or direct as the decision process in educational aspiration is complex, right from access, participation, and success.

However, there is limited understanding of the interplay of these factors on students’ college decisions. Harris and Halpin’s (2002) model on factors influencing post-secondary education (FIPSE) categorizes the factors into different domains, including educational processes, and personal, social, and cultural factors. The model has been adapted for various cultures to assess the factors affecting students’ higher education decisions, such as for examining first-generation college students (e.g., Coy-Ogan, 2009). It was nevertheless imperative to adapt the instrument to suit the social fabric of Hong Kong society. In achieving the research objectives, this study examined the dynamic interplay between personal and contextual factors affecting the choice of students from diverse backgrounds in higher education pursuits (Hossler et al., 1999).

### 1.3 Access to higher education in Hong Kong

Following the internationalization and rapid expansion of PSE, the number of PSE students rose from 25.8 percent in 2010 to 34.5 percent in 2020 (Census and Statistics Department, 2021). The sharp increment has escalated educational stratifications across institutions and privileged and underprivileged student groups and widened social and educational inequalities (Lam et al., 2019; Wong, 2021). Both 2016 and 2011 census data have shown that over 48 percent of students enrolled in competitive research-intensive institutions came from the top 10 percent of the wealthiest families. Over 30 percent of students enrolled in self-financed, private/vocational-oriented institutions lived below the poverty line. The clientele of self-financed institutions is mainly from low-income families, and they anticipate heavy student loans, keen competition from non-local students, and dim employment prospects upon graduation (Yip and Peng, 2018). The PSE expansion is driven by a neoliberal ideology (Lo, 2017), celebrating the free-market economy, and survival of the fittest (Lo, 2017; Wong, 2021). Only a small number of elite students from community colleges are able to transfer to the more resourceful government-funded universities. This leaves many less affluent students, who invested heavily in their second chance at higher education, without any certainty about the economic returns of their bachelor’s degree (Wong, 2021). Additionally, due to their inadequate proficiency in Chinese Language, the majority of ethnic minority South Asian students are unable to study beyond senior secondary education in Hong Kong (Hong Kong Unison, 2018).

Segmented educational pathways of under-represented groups such as low-income mainstream and ethnic minority students have led to stratified educational and career outcomes (Yip and Peng, 2018). The social equity and educational equality gaps in higher education indicate a necessity for collaborative institutional research, leveraging the synergies of various researchers to construct a comprehensive understanding of higher education in Hong Kong. However, there remains a dearth of research concerning the educational experiences and well-being of higher education students from diverse socio-cultural backgrounds, particularly during their initial and transitioning years of college (Gao, 2017; Yuen et al., 2021). Recent findings as reported by Yuen et al. (2021) and Yuen (2022), reveal that underprivileged Chinese immigrant and South Asian students are confronted with more obstacles and fewer opportunities before, during, and after their higher education, in comparison to their more affluent mainstream Chinese peers.

Against this backdrop, this study first seeks to ascertain significant factors that influence the aspirations of senior secondary students in Hong Kong toward higher education, including average and low-income mainstream, Chinese immigrant, and South Asian students. Second, the study examines the interactional effects of these factors and, third, delineates how diverse groups of students are impacted by them.

Consistent with the objectives, the study has three research questions:

(a) What factors impact students’ aspirations and pursuits in higher education? And which of them has the most significant impact?
(b) How do these factors interact with each other, directly or indirectly?
(c) What are the differences in higher education aspirations and pursuits among South Asian and Chinese immigrant students and local Hong Kong students from low and average income families?

## 2 Materials and methods

### 2.1 Participants and procedures

To examine the characteristics of the study participants, a purposive stratified sampling technique was utilized. Secondary schools were chosen based on two criteria: (1) having a minimum of 20% representation of a particular student group, such as low-income mainstream, Chinese immigrant, or South Asian students, and (2) being located in different districts across Hong Kong, Kowloon, and the New Territories. The selected schools were sent formal invitations and consent forms, and students had the option to decline participation while maintaining confidentiality. The Survey and Behavioral Research Ethics Committee of the lead author’s university granted approval. The study included a total of 4,850 students from grades 10–12 in 23 secondary schools, with the sample demographics shown in Table 1.

**TABLE 1:** Demographic profiles of students in each student group.

Student group	South/Southeast Asian students	Chinese immigrant students	Low-income HKMS	Average HKMS
Gender				
Male	355 (47.7%)	352 (52.4%)	515 (47.5%)	954 (49.4%)
Female	390 (52.3%)	320 (47.6%)	570 (52.5%)	977 (50.6%)
Age [Mean (SD)]	16.52 (1.24)	17.44 (1.26)	16.52 (1.15)	16.49 (1.13)
SES				
Subsidized	183 (26.6%)	279 (46.3%)	1086 (100%)	0 (0%)
Non-subsidized	506 (73.4%)	324 (53.7%)	0 (0%)	1942 (100%)
Mother’s highest education level				
Secondary or below	535 (72.5%)	570 (87.0%)	974 (91.2%)	1534 (80.4%)
Post-secondary or above	203 (27.5%)	85 (13.0%)	94 (8.8%)	374 (19.6%)
Father’s highest education level				
Secondary or below	505 (69.6%)	535 (81.8%)	956 (91.5%)	1438 (75.9%)
Post-secondary or above	221 (30.4%)	119 (18.2%)	89 (8.5%)	456 (24.1%)
Religion				
Religious	692 (92.51%)	137 (20.6%)	775 (71.8%)	595 (31.0%)
Non-religious	56 (7.49%)	528 (79.4%)	304 (28.2%)	1325 (69.0%)
Academic performance				
English				
Pass	694 (93.8%)	342 (51.4%)	720 (67.2%)	1408 (73.2%)
Fail	46 (6.2%)	324 (48.6%)	352 (32.8%)	516 (26.8%)
Chinese				
Pass	621 (83.4%)	598 (89.7%)	906 (84.4%)	1568 (81.5%)
Fail	124 (16.6%)	69 (10.3%)	168 (15.6%)	355 (18.5%)
Mathematics				
Pass	456 (62.1%)	555 (83.5%)	767 (71.5%)	1426 (74.1%)
Fail	278 (37.9%)	110 (16.5%)	306 (28.5%)	498 (25.9%)

The sample size for SAS, CIS, Low-income HKMS, and Average HKMS is 753, 673, 1086, and 1942, respectively. The data shown in the table is calculated by excluding unreported cases.

### 2.2 Measurement of variables

As indicated previously, this study developed and validated the Post-Secondary Education Pursuit Instrument (PSEPi) to assess the success and obstacle factors influencing students to pursue higher education in Hong Kong. Seven factors that can impact this pursuit, namely, significant others’ influence, glass ceiling effect, locus of control, self-efficacy, relative functionalism, preparation for college, and financial aid, were established. In addition, the PSEPi also considers demographic information, including age, gender, grade level, ethnicity, religious affiliation, recipient of the School Textbook Assistance Scheme, and parental occupation and education, as independent variables.

#### 2.2.1 Item selection

The items included in this study were selected through a rigorous examination of relevant literature, empirical research, and policy documents. While certain scales such as the college-going self-efficacy scale and perceptions to barriers scale have been previously published, their psychometric properties were not reported (Fore and Chaney, 1998). Consequently, these scales were not utilized in the present study.

Utilizing Harris’s (2015) scale, we developed a culturally and educationally specific questionnaire for the context of Hong Kong. Following international practice (Yuen and Lee, 2013), the scale was translated into Chinese and back-translated to cater to Chinese students. Based on literature and student interviews, we adapted the scale to fit the Hong Kong context. Our questionnaire focused on five key factors in FIPHE, namely relative functionalism, the glass ceiling effect, self-appraisal, preparation for college, and financial aid concerns. To identify the most influential person in a student’s pursuit of PSE, we designed the questionnaire to inquire about significant others’ impact, using four items for this purpose. The questionnaire can be found in Appendices 1 and 2. Students were asked to respond on a 4-Likert point scale (1 = strongly disagree to 4 = strongly agree) for all subscales except for the significant others’ influence subscale, which is in a 4-Likert point (1 = never to 4 = often), and preparation for college subscale, which is in a 2-Likert point scale (1 = false; 2 = true). The construct validity of PSEPi for Hong Kong youth has been supported (Chi-square (χ^2^) of 959.5 with 413 degrees of freedom (df), *p* < 0.001, NNFI = 0.98, CFI = 0.99, RMSEA = 0.042 and SRMR = 0.056). The composite reliability of the subscales is good (from 0.75 to 0.93).

### 2.3 Analytical strategies

This study aims to identify the factor that has the most influence on students’ educational aspirations by examining the construct validity of PSEPi and comparing the means of different factors in each group. To conduct the analysis, the CFA model was analyzed with LISREL 8.54 using diagonally weighted least squares (DWLS) estimation, treating the data as ordinal (Mîndrilã, 2010). The measurement models were evaluated using several fit indices, including Root Mean Square Error Approximation (RMSEA), Standardized Root Mean Square Residual (SRMR), Comparative Fit Index (CFI) and Non-Normed Fit Index (NNFI), in conjunction with chi-square test statistics. To measure the model fit, we employed a set of criteria, with NNFI and CFI values greater than 0.90 and >0.95 indicating a good fit (Kline, 2011), and an RMSEA value smaller than 0.05 and SRMR smaller than 0.08 considered adequate (Hu and Bentler, 1999). The results showed that the relative functionalism factor had the highest means and, thus, exerted the most significant impact on students’ pursuit of higher education across all groups.

To determine how various factors affect students’ pursuit of PSE and how these factors interact with relative functionalism, directly or indirectly, the structural equation modeling (SEM) techniques were utilized to perform direct effects models and multiple mediational models. The direct effects models involved using six correlated success factors and obstacles simultaneously to predict the relative functionalism of each student group. Each latent factor was predicted by its corresponding indicators, with uncorrelated uniqueness specified among the indicators. In the multiple mediational models, specific attention was given to the influence of significant others and locus of control as independent variables and how they affect relative functionalism as dependent variables, with the remaining factors serving as mediators. Finally, one-way MANOVA was conducted to explore the perceived differences in the factors affecting students’ aspirations and pursuit of higher education among different student groups.

## 3 Results

### 3.1 Factorial validity of PSEPi in different student groups

Table 2 presents the factorial validity of the seven-factor model of the adapted questionnaire instrument across all groups of students. As all values of NNFI and CFI were greater than 0.95, and all values of RMSEA were equal or less than 0.050, the seven-factor model of PSEPi fitted the data of each student group well. Seven conceptually distinct factors, namely, significant others’ influence, glass ceiling, relative functionalism, locus of control, self-efficacy, preparation for college, and financial aid were identified in each student group. The mean score for relative functionalism was the highest, ranging from 3.12 to 3.34 out of 4.00, among all the factors in each student group.

**TABLE 2:** Goodness-of-fit indices of the seven-factor model of PSEPi and factor means in each group.

	CIS	SAS	Low-income HKMS	Average HKMS
1. Goodness of fit indices				
χ^2^	2561.33	2496.74	3817.13	4955.43
df	413	413	413	413
*p*-value	0.001	0.001	0.001	0.001
NNFI	0.98	0.98	0.97	0.97
CFI	0.98	0.98	0.97	0.98
RMSEA	0.042	0.041	0.050	0.044
SRMR	0.062	0.060	0.058	0.052
2. Factor means (SD)				
Significant others influence	2.95 (0.03)	3.09 (0.03)	2.81 (0.02)	2.85 (0.02)
Glass ceiling	2.29 (0.02)	2.56 (0.02)	2.25 (0.02)	2.14 (0.01)
Relative functionalism	3.24 (0.02)	3.34 (0.02)	3.16 (0.02)	3.12 (0.01)
Locus of control	2.94 (0.02)	2.98 (0.02)	2.90 (0.02)	2.89 (0.01)
Self-efficacy	2.70 (0.02)	2.98 (0.02)	2.58 (0.02)	2.59 (0.01)
Preparation for PSE	0.47 (0.33)	0.49 (0.33)	0.43 (0.31)	0.43 (0.31)
Financial aid	2.44 (0.02)	2.64 (0.02)	2.48 (0.02)	2.31 (0.01)

χ2, chi-square; df, degree of freedom; NNFI, non-normed fit index; CFI, comparative fit index; RMSEA, root-mean-square error of approximation; SRMR, standardized root-mean-square residual.

### 3.2 Predictions for relative functionalism among different student groups

Table 3 illustrates the significant direct effects of the significant others, locus of control and self-efficacy factors on the relative functionalism (utilitarian educational values) of students across all groups (*p* < 0.05). Particularly for Chinese immigrant students and low-income Hong Kong mainstream students groups, all factors were significant predictors (βs ranged from −0.10 to 0.43 in Chinese immigrant student group while from −0.07 to 0.28 in low-income Hong Kong mainstream student group) with the locus of control (βs equaled 0.43 and 0.28 in Chinese immigrant and low-income Hong Kong mainstream student groups, respectively) being the most important contributor (*p* < 0.05). For South Asian students, all factors, except for the glass ceiling factor, were significant predictors (βs ranged from −0.18 to 0.52) (*p* < 0.05) and among which the locus of control and significant others were the most important contributors. Average Hong Kong mainstream students have a significant effect on the locus of control, significant others, and self-efficacy (βs ranged from 0.17 to 0.37) (*p* < 0.05). To summarize, significant others and locus of control were the common positive predictors of relative functionalism, across all cultural backgrounds. Financial aid and preparation for college were essential predictors of relative functionalism for all underprivileged groups, except for the average Hong Kong mainstream students.

**TABLE 3:** SEM Direct effects model examines the relationships between the six factors and relative functionalism across groups.

	Relative functionalism			
	Chinese immigrant students	South/Southeast Asian students	Low-income Hong Kong mainstream students	Average Hong Kong Mainstream students
Significant Others’ Influence	0.30*	0.31*	0.06*	0.21*
Glass Ceiling	0.17*	−0.01	0.08*	0.01
Locus of Control	0.43*	0.52*	0.28*	0.37*
Self-efficacy	0.19*	−0.18*	0.19*	0.17*
Preparation for PSE	0.14*	−0.10*	−0.07*	0.01
Financial Aid	−0.10*	0.24*	0.10*	0.02

*p < 0.05.

### 3.3 Mediational models between significant others and locus of control, and relative functionalism

The direct effects analyses revealed that the significant others and locus of control factors were the main contributors to the relative functionalism of students in all groups. Deliberations were made to examine whether other factors (e.g., glass ceiling effect, self-efficacy, locus of control, preparation for college, and financial aid) transmit the effect of the influence of significant others on the relative functionalism among the mainstream, immigrant, and minority groups of students. Likewise, the interplay between locus of control and other factors on educational functionalism beliefs was further explored. A multiple mediation model using significant others as an independent variable, other factors like self-efficacy as mediators, and relative functionalism as a dependent variable was specified and analyzed across each group. In a similar vein, another multiple mediation model was conducted using the locus of control factor as an independent variable, other factors like preparation for college as mediators, and relative functionalism as a dependent variable across all groups of students. Table 4 illustrates the good fit of the multiple mediational models. All values of NNFI and CFI were higher than 0.95 and all values of RMSEA were equal or less than 0.050. Both significant others and locus of control factors were critical mediators with significant mediating effects (βs ranged from 0.05 to 0.12) (*p* < 0.05), which had noticeable impacts on the relative functionalism belief among different groups of students. The findings also revealed their reciprocal relationships in determining the relative functionalism beliefs of students from diverse backgrounds.

**TABLE 4:** SEM – Fit indices and standardized total direct and specific indirect effects of multiple mediation analyses.

Multiple mediation models	Total direct effect (95% CI)	Specific indirect effect (95% CI)	χ2 (df)	NNFI	CFI	RMSEA	SRMR
**CIS**							
Locus of control → Significant others’ influence → Relative functionalism	β = 0.38* (0.18, 0.58)	β = 0.07* (0.02, 0.12)	2561.33(413)	0.98	0.98	0.042	0.062
Significant others’ influence → Locus of control → Relative functionalism	β = 0.23* (0.11, 0.35)	β = 0.12* (0.01, 0.23)	2561.33(413)	0.98	0.98	0.042	0.062
**SAS**							
Significant others’ influence → Locus of control → Relative functionalism	β = 0.34* (0.15, 0.53)	β = 0.07* (0.02, 0.12)	2496.74(413)	0.98	0.98	0.041	0.060
**Low-income HKMS**							
Significant others’ influence → Locus of control → Relative functionalism	β = 0.10 (−0.02, 0.22)	β = 0.07* (0.02, 0.12)	3817.14(413)	0.97	0.97	0.050	0.058
**Average HKMS**							
Locus of control → Significant others’ influence → Relative functionalism	β = 0.29* (0.18, 0.40)	β = 0.05* (0.01, 0.09)	4955.43(413)	0.97	0.98	0.044	0.052
Significant others’ influence → Locus of control → Relative functionalism	β = 0.18* (0.13, 0.23)	β = 0.08* (0.03, 0.13)	4955.43(413)	0.97	0.98	0.044	0.052

Sobel tests were applied for total, direct and specific indirect effects. 95% confidence intervals were also reported. *p < 0.05.

### 3.4 Group differences in the seven factors of PSEPi among student groups

To assess the differential impacts of the salient factors affecting higher education pursuits across groups of students, a one-way MANOVA test was administered. Table 5 reveals significant student group differences in seven factors associated with educational aspirations. South Asian students scored higher than average Hong Kong mainstream students in all factors (*p* < 0.05) (Cohen’s *d* values ranged from 0.16 to 0.72). Similarly, Chinese immigrant students scored higher than average Hong Kong mainstream students in all factors (*p* < 0.05) (Cohen’s *d* values ranged from 0.13 to 0.27), except for the locus of control and preparation for college. There was a slight difference between low-income Hong Kong mainstream students and average Hong Kong mainstream students; the former scored higher on two obstacle variables, the glass ceiling (*d* = 0.20) and financial aid (*d* = 0.28).

**TABLE 5:** MANOVA – Mean comparisons of the Low-income HKMS, CIS, and SAS against Average HKMS.

Reference group:	average HKMS	Significant others	Glass ceiling	Relative functionalism	Locus of control	Self-efficacy	Preparation for PSE	Financial aid
Low-income HKMS	Mean differences	−0.04 (−0.06)	0.11*(0.20)	0.04 (0.08)	0.01(0.02)	−0.01 (−0.02)	0.00 (0.01)	0.17*(0.28)
CIS	Mean differences	0.10*(0.13)	0.15*(0.27)	0.12*(0.23)	0.05(0.09)	0.11*(0.19)	0.04(0.12)	0.13*(0.21)
SAS	Mean differences	0.23*(0.33)	0.42*(0.72)	0.22*(0.41)	0.09*(0.16)	0.39*(0.65)	0.06*(0.19)	0.33*(0.53)

Effect size (Cohen’s d) is indicated in the parenthesis. (0.2 small, 0.5 medium and 0.8 large) *p < 0.05.

## 4 Discussion

This study deepens our understanding of the differential impacts of various factors on higher education beliefs and aspirations across different groups of students in Hong Kong. Along with international studies (e.g., Hossler et al., 1999), the findings confirmed that the college and university enrollment gaps between local Hong Kong, underprivileged Chinese immigrant, and South Asian students correlated to multiple personal and contextual factors with differential effects. The Confirmatory Factor Analysis results revealed that the adapted seven-factor questionnaire was sensitive and valid to the Hong Kong study. Particularly, students’ belief in the utilitarian function of higher education (relative functionalism) is the most critical factor in determining their aspirations and pursuits of higher education. The findings aligned with international studies (e.g., Sier, 2021; Bataeineh, 2022; Vietze et al., 2022), and suggested that instilling a sense of meaning and hope in students can encourage them to pursue further studies.

While students’ belief in the values of higher education is influenced by their parents, teachers and peers, the study confirmed that socio-demographic and socioeconomic factors interact to impact students’ beliefs in the function of education. Consistent with previous studies (e.g., Chiu and Walker, 2007) this study delved deeper into the impacts of the mechanism of the glass ceiling effects among the disadvantaged mainstream, Chinese immigrant, and ethnic minority South Asian students and their advantageous mainstream peers. Despite recognizing the value of higher education for personal advancement, systemic barriers associated with the lack of social and economic capital, language proficiency and self-efficacy in pursuing higher education led these students to be trapped in the vicious cycle of early dropout, low educational aspirations, and entry into the labor market without marketable qualifications (Bhowmik, 2019; Lam et al., 2019; Chiu and Siu, 2022).

### 4.1 Intersectionality confronted by underprivileged students

Our direct effects analyses confirmed that significant others’ influence and locus of control were salient factors facilitating the higher education aspirations of South Asian and Chinese immigrant students. These findings affirm the studies (Wang and Neihart, 2015; Lam et al., 2019; Keung and Ho, 2020) that family socio-economic status and personal capability are dominant factors affecting their education aspirations. Hong Kong is a collective Asian society, parents and teachers are the youth’s dream keepers and life anchors. Family influence and locus of control accounted for the most variance in mitigating the financial barriers of Chinese immigrant students’ higher education pursuits. Compared to economic factors, parental care and educational aspirations for a brighter future are the essential student-centered enablers in promoting their resilience and self-efficacy (Hossler et al., 1999). To this end, school personnel need to recognize the systemic barriers different student groups confront (Yuen et al., 2021), give academic planning advice, and address such immigrant family concerns by tackling different barriers.

By contrast, the glass ceiling factor links with relative functionalism among Chinese immigrant and low-income mainstream students, suggesting it to be a common obstacle with heterogeneous items in these two student groups. The glass ceiling factor encompasses a range of issues, including family backgrounds, ethnic culture, financial situation, language ability, lack of expert advice and guidance, and societal expectations on gender. This study is in parallel to prior international research (Hossler et al., 1999; Harris and Halpin, 2002; Chowdry et al., 2011; Ding et al., 2021) by elucidating the various hurdles that underprivileged mainstream, Chinese immigrant, and ethnic minority scholars encounter while pursuing higher education. Factors such as family’s socioeconomic status, ethnic customs, and societal gender norms can pose significant challenges, particularly for female students from immigrant and ethnic minority backgrounds who aspire to higher education. Given the educational segregation and stratification of these student groups in higher education, this study presents the latest evidence of how the complex social and cultural capital dynamics play out. On the one hand, social and economic vulnerabilities may prevent these students from applying to university due to their lack of academic readiness, and cultural and social capital. On the other hand, underprivileged Chinese immigrants and South Asians value higher education and have high expectations for their future earnings and life chances. These beliefs and aspirations are deeply rooted in Confucian culture and Asian family values (Ho et al., 2018; Yuen, 2022). Although South Asian students face language and financial barriers, they attribute higher utilitarian values to university degrees than their mainstream peers in order to mitigate their disadvantageous social circumstances and achieve upward mobility. The prevalent Hong Kong government policy measures are narrowly concentrated on remediating the limited Chinese proficiency of South Asian students, with little attention paid to enhancing the English proficiency of Chinese immigrant students. A comprehensive social and educational policy is needed to enable success for all students, regardless of their cultural backgrounds. This should include measures such as quality parent-child relationships, raising agentive and educational aspirations, and targeted higher education subsidies.

### 4.2 Parents, self-beliefs and higher education pursuit

Delving deeper into the intricate relationship between various factors and their influence on students’ belief in the functional values of higher education, the mediation analysis highlighted the pivotal role of the locus of control factor in mediating the impacts of significant others on relative functionalism. It is evident that students’ parents, friends and teachers directly affect their belief in the value of higher education, along with themselves. Notably, Asian parents are their children’s dream keepers and exert an affirmative mindset on their commitment to higher education pursuits. Moreover, the reciprocal dynamic between parents and students plays a critical role in determining relative functionalism. The evident family influence calls for functional home-school collaboration in high schools to prepare, advise, and support underprivileged mainstream and immigrant students to enhance their locus of control and self-efficacy. Strong family involvement is part of school capital in mitigating the glass ceiling effect caused by adverse social conditions (de Lugt, 2020; Yuen et al., 2021).

The influence of parents on their children’s educational aspirations and the sense of control was also illustrated. The family serves as a perennial institution for mediating the negative or unproductive mindset and increases youth self-efficacy and agency in accepting disappointments, challenges, and unpredictable changes in life (Patfield et al., 2021). The roles of parents and teachers in students’ educational aspirations have been well-documented (Ng and Choo, 2021; Xiang and Chiu, 2022). This study underscored that the mothers are the primary caregivers and, in most cases, account for the greatest explanatory power for children’s desire to pursue higher education. Parents are valuable community partners, albeit they represent a highly heterogeneous group in terms of education, socio-economic status, linguistics, and cultural traditions. Governments should provide specific education for parents to help foster their children’s aspirations for higher education and boost academic confidence (Gamble and Crouse, 2020). This also calls for further deliberations from the government to transform the power of familial habitus for underprivileged students whose parents have no knowledge of higher education but have high expectations of their future. Additionally, students who possess a strong sense of internal control are more likely to take responsibility for their academic decisions, seek guidance actively, and ultimately succeed in achieving their higher education goals (Nordstrom and Segrist, 2009).

Contrary to a common belief, although echoing prior studies (Yuen et al., 2021), local students in Hong Kong have more reservations about the value of their higher education credentials in terms of advancing their academic and career goals compared to their non-local peers. This suggests that they may be disillusioned about the future. The negative attitudes toward university education in Hong Kong among local students may result from witnessing emigration following the social unrest in 2019. The survey was conducted between late 2018 and mid-2019 during a period of major social conflicts between local and non-local students in higher education institutions. This was further compounded by increasing competition from cities in the Greater Bay Area of mainland China.

In comparison to their typical Hong Kong mainstream counterparts, low-income Hong Kong mainstream students rated all factors as less significant. Despite being local, poor mainstream students do not face the ethnic acculturation challenges they have limited aspirations for upward social mobility. The segmented pathways in higher education coupled with the competitive job market do not promise marketable qualifications (Wong, 2021). The government is obliged to address this unacceptable social inequality. Similar to other regions (Davies and Ercolani, 2021), negative beliefs and perceptions about higher education may lower the motivation of local students and perpetuate existing educational stratifications and segmented pathways for struggling students.

The glass ceiling effect is particularly prevalent among South Asian students due to financial hardship, language constraints, limited social networks, and inadequate higher education guidance services. Such intersection coupled with a self-reliant discourse under the impacts of neoliberalism only perpetuates the prevalent systemic barriers to achieving meaningful higher education dreams. A recent study revealed that South Asian students exhibited the most profound difference from Hong Kong mainstream students concerning language proficiency, ethnic culture, self-efficacy, and knowledge of financial aid (Yuen et al., 2021). Prior research (Gao et al., 2019; Yuen and Leung, 2019; Loh and Hung, 2020; Yuen et al., 2021; Yuen, 2022) has established that South Asian students perceive their ethnic culture, lack of Chinese proficiency, and gender roles as obstacles to pursuing higher education, which ultimately lowers their aspirations.

That is said, it is evident that students’ beliefs are drivers of their pursuit of higher education. A positive social image and empowered self-agency can help mitigate their disadvantage. As revealed by the findings, Chinese immigrant students scored higher in success factors (significant others influence, relative functionalism, and self-efficacy) and barriers (glass ceiling and financial aid) than the average Hong Kong mainstream students. They face comparable obstacles to higher education as South Asian students, albeit to a different extent. Despite facing fewer ethnic and language barriers, Chinese immigrant students frequently balance work and study to support their families and be academically self-reliant (Yuen and Leung, 2019). Future research should examine the interplay between personal resilience and the influence of significant others to comprehend the mechanisms underlying the structural relationships among the factors.

The findings by direct effects and mediation analyses call for tailored guidance plans involving home-school collaboration, self-advocacy skill development, and emotional and mental support, which are necessary to address their diverse educational journeys. Increasing local students’ hope for the future deserves further investigation as they are the future pillar of society. At the core of higher education is talent development. Policy-makers are concerned with producing efficient and competitive citizens and the labor force for economic development. Efforts to improve access to higher education for disadvantaged youth should focus on creating nested and genuine home-school-community partnerships to empower parents to provide concrete support and advice to their children regarding matching their abilities, career aspirations, and needs. Implications for restructuring the hierarchy of the higher education system (Xiang and Chiu, 2022) include broadening the choice of specialization programs for different ethnic student groups to promote both vertical and horizontal development. Additionally, cultural-specific parental education programs can be developed to align parental expectations with college preparation strategies.

### 4.3 Limitations and future research

Our research methodology dictates our findings. The cross-sectional findings can be used as a starting point for future studies on higher education trajectories of diverse Hong Kong youth over time. However, it is important to note that this study cannot establish a direct causal effect relationship between each factor and a student’s decision to pursue higher education. The findings should be interpreted within a specific context and time period. Hong Kong has turned a new chapter after the 2019 social unrest and the COVID-19 pandemic. The higher education options available to middle-class and working-class families, as well as mainstream or non-mainstream students, vary greatly. The former navigated their children to diversified overseas education pipelines while their working-class counterparts struggled to support their children by applying for local self-financed higher education programs. To gain a deeper understanding of the educational pathways of students from diverse cultural backgrounds, future research should adopt a longitudinal design. It is worth noting that only self-reported data were collected and utilized for analysis, which may have introduced some bias that could affect the validity of the findings. To overcome this issue in the future, we suggest employing multiple research methods that can incorporate both quantitative and qualitative data. Lastly, it is important to keep in mind that the sample size of each student group was not equal. We had fewer socially disadvantaged students than the average mainstream students. Hence, our findings should be interpreted with caution, and future research should make an effort to recruit a larger proportion of socially disadvantaged students.

## 5 Conclusion

This research delves into the complex issue of segmented educational pathways for underprivileged students in pursuing higher education. It examines the various contextual and demographic factors that influence a student’s decision-making process, taking cultural and socio-economic insights into account. The study highlights the divergent values placed on higher education by mainstream and non-mainstream, privileged and underprivileged youth in Hong Kong. Parental care and a student’s inner strengths and self confidence are critical factors that significantly impact a student’s likelihood of pursuing higher education. Additionally, the language barrier (primarily academic English) faced by low-income mainstream students remains the crux of the problem for pursuing higher education. This issue, along with financial aid, requires language-focused initiatives from the government to improve English language proficiency rather than letting these youth bear the impacts of structural inequalities imposed by the neoliberalism of higher education. Economic capital is a critical factor in educational aspirations, and designated financial assistance schemes can alleviate the financial burden on low-income families. The findings of our research demonstrate that a student’s educational beliefs and aspirations are positively impacted by their locus of control and the influence of significant others. Nevertheless, it is imperative to acknowledge that the effects of these factors can vary among diverse student populations. Thus, it is crucial to avoid applying a blanket solution to underprivileged student groups and instead recognize the distinctiveness within and between these groups. Policy makers must consider the unique socio-cultural context of Hong Kong when designing interventions aimed at promoting social inclusion and addressing the exclusion of disadvantaged students. It is noteworthy that this issue is not exclusive to Hong Kong but is prevalent in other societies where underprivileged groups experience comparable systemic discrimination in higher education. Our study sheds light on the intersectionality issues encountered by underprivileged youth in education, providing potential avenues to promote equity in secondary and higher education.

## Data availability statement

The datasets presented in this article are not readily available because the ethics approval for this study has restricted the sharing of data outside of the immediate research group. Requests to access the datasets should be directed to CY at yuetmuiyuen@cuhk.edu.hk.

## Ethics statement

The studies involving humans were approved by the Survey and Behavioural Research Ethic Committee. The studies were conducted in accordance with the local legislation and institutional requirements. The participants provided their written informed consent to participate in this study.

## Author contributions

CY: Conceptualization, Data curation, Formal analysis, Funding acquisition, Investigation, Methodology, Project administration, Supervision, Writing – original draft. AC: Methodology, Writing – review and editing. KL: Data curation, Formal analysis, Methodology, Writing – review and editing.
